# Branching-scenario games in acute end-of-life care education: cultural adaptation and health equity from a public health implementation perspective

**DOI:** 10.3389/fpubh.2026.1889780

**Published:** 2026-06-18

**Authors:** Fanyi Cheng, Tao Xu, Junwei Mao, Zhiping Li, Yajun Wang

**Affiliations:** Department of Emergency Medicine, People's Hospital of Quzhou, Quzhou, China

**Keywords:** branching-scenario games, cultural adaptation, death literacy, end-of-life care, health equity, palliative care education, public health implementation, serious games

## Abstract

The quality and accessibility of end-of-life (EOL) care are sensitive indicators of health equity, yet these services remain profoundly unequal worldwide. The challenge is especially acute in emergency and critical care, where clinicians must reconcile time-pressured medical decisions with emotionally charged communication. Branching-scenario games, serious games built on clinical decision trees, have emerged as a promising educational tool for rehearsing such competencies in a safe, repeatable virtual environment. However, their effectiveness is not a matter of simple technology transfer: it depends heavily on cultural fit and on an explicit commitment to health equity. In this mini-review, we examine branching-scenario games in acute EOL care education through a public health implementation lens. We synthesise evidence on their value and limitations, review principles and practise of cultural adaptation, situate them within debates on health equity and barriers to equitable care, and consider how they might be scaled from discrete educational projects to system-level public health interventions. We then summarise academic controversies, identify research gaps, and outline future directions, including participatory design, mixed-methods evaluation, and AI-driven adaptive learning. We argue that, with rigorous cultural adaptation and equity-by-design, these games can evolve from a novel teaching technology into a lever for advancing equitable EOL care.

## 1 Introduction

The quality and accessibility of end-of-life (EOL) care are sensitive indicators of how equitably a society distributes health. Yet globally, these services remain profoundly unequal, with only about 14% of the worldwide need currently met (1). The challenge is especially acute in emergency and critical care settings, where clinicians must reconcile complex, time-pressured medical decisions with emotionally charged human communication. Conventional didactic models struggle to cultivate the core competencies, particularly communication skills and cultural sensitivity, that such high-pressure encounters demand.

Against this backdrop, simulation-based teaching, and branching-scenario games in particular, has begun to enter EOL care education. Unlike linear simulations, quiz- or drill-based games, or open-ended virtual-patient and role-play environments, branching-scenario games are built on an explicit clinical decision tree: each choice opens a distinct narrative pathway with its own consequences, so that the link between a clinician’s communication and management decisions and their downstream outcomes becomes unusually visible and rehearsable. By embedding decision trees in realistic clinical situations, these games let learners explore the consequences of different choices in a safe virtual space, accumulating experience and confidence without risk to patients (2). Such “serious games” have been shown to improve clinicians’ knowledge, skills, and self-efficacy, and appear well suited to complex competencies such as communication and ethical decision-making (3, 4). Deploying them in acute EOL care, however, is not a matter of simple technology transfer. Their effectiveness depends heavily on whether the content authentically reflects the clinical dilemmas faced by the target learners and the cultural, social, and systemic forces underlying those dilemmas. One study found that, despite substantial cultural differences, a US-derived serious-illness communication tool could be effectively adapted to an African context through co-creation with local practitioners (5), a reminder that cultural fit, more than technical sophistication, determines whether an educational intervention succeeds.

This mini-review, therefore, examines branching-scenario games in acute EOL care education through a public health implementation lens, focusing on two intertwined themes: cultural adaptation and health equity. We summarise their value and limitations, review adaptation principles and practise, situate them within debates on health equity and systemic implementation, identify research gaps, and outline future directions, to inform more inclusive and effective models of EOL care education.

## 2 Value and challenges in acute EOL care education

The principal value of branching-scenario games lies in offering a safe, repeatable rehearsal environment. A study of emergency-medicine residents using a “LIVE. DIE. REPEAT.” branching format reported significant gains in confidence around EOL communication and palliative management (2). Learners felt better able to respond to clinical deterioration and to make assessments and decisions. The post-game debrief was regarded as pivotal, promoting self-reflection and consolidation of learning (2). In nursing education, virtual clinical simulation has similarly improved students’ knowledge, competence, and attitudes toward EOL care, serving as a valuable supplement where clinical placements are scarce (6). A systematic review likewise concluded that gaming and game-based learning hold promise in palliative care education, especially for creating a safe learning environment (4). Much of this engagement is driven by core gamification mechanics, structured feedback, progressive challenge, scoring, and visible markers of mastery, that reward sound clinical reasoning and motivate repeated practise. In acute EOL care, however, these rewards must be tied to process quality, such as the conduct of difficult conversations and shared decision-making, rather than to “winning” against death, so that the reward structure reinforces rather than trivialises the gravity of the subject (Table 1).

**Table 1:** Evidence-to-implementation framework for culturally adapted, equity-oriented branching-scenario games in acute end-of-life care education.

Evidence-to-implementation domain	Representative evidence	What the evidence indicates	Branching-scenario design requirement	Public health and equity priority
Educational effectiveness	Stanich et al., 2023 (2); Zhang et al., 2024 (6)	Gamified or virtual simulation can improve EOL-related confidence, knowledge, competence, and attitudes in professional learners.	Use realistic deterioration and communication decisions with structured feedback and facilitated debriefing.	Move beyond learner satisfaction to assess communication behaviour, decision quality, and potential effects on care experience.
Evidence quality and evaluation	Peek et al., 2026 (4)	Evidence is promising but heterogeneous, often small-scale, and insufficiently theory-driven.	Specify active ingredients, such as narrative immersion, feedback, role perspective, and repeated practise.	Adopt mixed-methods evaluation and implementation outcomes before population-level scale-up.
Deep cultural adaptation	DeBoer et al., 2026 (5); Dai et al., 2025 (9)	Effective transfer requires iterative local co-design and adaptation of underlying norms, not translation alone.	Co-design dialogue, family roles, emotional cues, and feedback pathways with local stakeholders.	Prevent culturally mismatched scripts from reinforcing inequitable communication or exclusion.
Values and advance care planning	Dupont et al., 2022 (10); Perin et al., 2022 (37)	EOL preference-elicitation tools require adaptation to local values, language, and legal context.	Embed preference elicitation and jurisdiction-sensitive treatment-limitation or ACP branches.	Support informed decision preparedness while respecting plural values and decision-making models.
Trust and minoritised communities	Anderson, 2021 (11); Chidiac et al., 2021 (28)	Culturally responsive education can address mistrust and strengthen inclusive palliative care competence.	Include scenarios involving mistrust, chosen families, identity affirmation, bias recognition, and respectful repair.	Design with affected communities so that equity is an explicit learning outcome rather than a peripheral theme.
Language justice and access	Bigger et al., 2025 (30); Dookie and Martin, 2025 (31)	Language discordance undermines palliative care quality and access; interpreter support is integral to equitable care.	Provide multilingual, low-literacy-compatible versions and simulate interpreter-mediated conversations.	Reduce digital, linguistic, and cognitive barriers that otherwise exclude underserved populations.
Death literacy and public engagement	Bollig and Bauer, 2021 (34); Macaden et al., 2022 (35); Bollig et al., 2022 (36)	Community education can improve public understanding of dying and palliative care across settings.	Create public- and caregiver-facing pathways for values clarification, ACP awareness, and family communication.	Position games as community education tools supporting death literacy and equitable preparedness.
Implementation and sustainable scale-up	Van Campe et al., 2025 (38); Wainwright et al., 2025 (40); Arendse et al., 2026 (44)	Scale-up depends on context-intervention fit, trusted connectors, training capacity, and local resources.	Separate core educational functions from adaptable cultural content and prioritise low-bandwidth delivery options.	Use implementation frameworks to monitor reach, acceptability, feasibility, sustainability, and equity of access.

Representative sources were selected from the mini-review to map direct educational evidence and transferable evidence on cultural adaptation, equity, and implementation; this table is not a systematic enumeration of all eligible interventions. ACP, advance care planning; EOL, end-of-life.

The challenges, however, are equally clear and span design, tone, resourcing, and evaluation. First, design quality directly determines educational impact. A poorly designed game may oversimplify reality or fail to reach the competencies that matter. Second, acute EOL care involves profound emotional and ethical dilemmas, and the “entertainment” element of gamification can sit in tension with the gravity of the subject (4). Balancing engagement against seriousness is a central design problem. Third, development is resource-intensive, requiring collaboration amongst clinicians, instructional designers, and technologists, a real barrier for under-resourced institutions and regions. Evaluation is also problematic. Most studies rely on learners’ subjective feedback (satisfaction, confidence) and lack objective measures of behaviour change in practise (3), and the existing evidence is of uneven quality and rarely theory-driven (4).

Most critically, the content, cases, and language of existing games are typically grounded in mainstream cultural assumptions, which may inadvertently entrench bias and overlook the needs of minority groups. A communication script rooted in Western individualism, for instance, may be ill-fitting or even counterproductive where family-centred decision-making prevails (7, 8). Converting these games from “universal” to “culturally specific” tools is thus a key step toward effectiveness in diverse settings.

## 3 Cultural adaptation: principles and practise

Cultural adaptation is not translation but a systematic, iterative, community-engaged process of redesign. Its guiding principle is to preserve the core active ingredients of an intervention while aligning its language, values, beliefs, social norms, and practises with the target population’s cultural context (9). Successful adaptation extends from “surface structure” (language, images, characters) to “deep structure” (cultural values, health beliefs, social context) (9).

Several studies offer transferable frameworks. An adaptation of the “Go Wish” card game for Flanders, Belgium, used an iterative process, repeated consultation with community organisations and ethnic and religious representatives, expert appraisal of linguistic equivalence, applicability, comprehensibility, and relevance, and final user testing (10); 16 of 36 cards were modified and 3 added to reflect local cultural and legal context (e.g., euthanasia legislation). An adaptation of a US serious-illness conversation guide in Rwanda followed a cultural-adaptation process model, using focus groups to identify local training needs, then piloting and refining the guide iteratively (5); this co-creation approach retained international best practise while honouring local norms.

The field’s applicability is broad, extending across national, ethnic, and faith communities. In mainland China, a basic palliative care curriculum underwent both surface- and deep-structure adaptation following Barrera’s framework and was successfully piloted with clinicians, who rated quality as high or very high (9). Adaptation for specific ethnic groups matters too. The “Let’s Talk About ACP” toolkit used culturally responsive methods to help African-American faith leaders teach their congregations about EOL options and advance care planning, explicitly addressing pervasive mistrust of the health system (11). In parallel, a “CASA” intervention for Latino families is combining meaning-centred psychotherapy with couples communication-skills training (12).

Together, these efforts reveal a key insight: effective adaptation must move beyond “adding cultural elements” to grasp a population’s underlying “cultural logic.” In Chinese contexts, where open talk of death is taboo and family decision-making often takes precedence over individual autonomy (7, 13), a successful game might design an indirect communication pathway that engages family members in shared decision-making to achieve “relational autonomy,” rather than confronting the patient directly (13). For Muslim patients, scenarios must respect specific religious rituals and family roles (14, 15).

## 4 A health-equity lens on educational intervention and barriers

From an equity perspective, a foundational goal of EOL care education is to eliminate disparities in care quality arising from race, ethnicity, sexual orientation, gender identity, language, and socioeconomic status. Yet current educational systems may reproduce or even amplify these inequities. LGBTQIA+ people face distinct barriers in EOL care, including clinician bias and discrimination, disregard for “chosen families,” and concealment of identity out of fear (16–18); a scoping review found their needs, experiences, and preferences to be largely neglected by existing research (17). For people of colour, especially African-American and Hispanic populations, historical mistrust, language barriers, and culturally insensitive services contribute to markedly lower utilisation than amongst White patients (19–22).

Education is a key lever, but its effect is blunted when content lacks cultural responsiveness. A survey of US hospice cultural-competence training found that, although most agencies offered such training, it concentrated on the largest minority groups and was assessed narrowly (e.g., post-session quizzes), without measuring real changes in clinical behaviour (22). More fundamentally, training often remains at the level of knowledge transfer (e.g., describing different death rituals) and fails to reach the deeper “structural competence” needed to recognise and counter the systemic discrimination, poverty, and social exclusion that drive inequity (23–25).

Branching-scenario games hold distinctive potential here. Carefully designed, they can simulate the discriminatory situations diverse patients may encounter and let learners experience and reflect on injustice in a safe space, for example, a scenario in which a transgender patient’s chosen family is ignored, or an African-American patient suffers unnecessarily from inadequate pain management (26, 27). Such immersion can evoke emotional resonance and cognitive shift more powerfully than lectures; a 1.5-h LGBTQ+ palliative care workshop significantly improved interdisciplinary teams’ knowledge, confidence, and comfort (28).

Barriers nonetheless persist at several levels. Many programmes still neglect LGBTQ+ needs (29). Language is another major challenge. A scoping review found that language discordance harms care quality and that qualified medical interpreters are central to equitable care (30, 31), and even the best-designed game cannot reach patients with limited English proficiency without versions in their own language. Low socioeconomic status and health literacy add further obstacles (32). A culturally adapted game that demands high technological access or reading ability may still exclude the most vulnerable. “Equity-by-design” principles, attending to language, educational level, technological access, and cognitive load, are therefore essential.

## 5 Public health implementation: from programmes to systems

Elevating branching-scenario games from discrete “educational projects” to “public health interventions” requires thinking at the system level about design, dissemination, sustainability, and impact. A public health view holds that EOL care should be a shared community and societal responsibility, not solely a task internal to health systems (33). Games can serve as a tool of “public health palliative care,” not only training clinicians but also educating the public and raising community “death literacy” (34, 35).

The “Last Aid” course is an instructive model. Originating in Germany, it uses community education to teach ordinary citizens the basics of death, dying, and palliative care so they can better support the dying around them (34); it has spread to more than 20 countries with cultural tailoring (36). Although not strictly a branching-scenario game, its community-empowerment ethos aligns closely with a public health implementation perspective. Games could likewise be designed as “community engagement tools” that let members explore death preferences and advance care planning, sparking public conversation about death at the community level (10, 37).

Moving from programme to system means crossing several barriers. First, robust implementation-science frameworks are needed: one project used the ADAPT guidance to transfer a Canadian community navigation programme to six European countries, stressing assessment of context–intervention fit, identification of core components, and collaboration with original developers (38), a methodological template for cross-cultural deployment. Second, sustainable “empowerment ecosystems” must be built, including training “community connectors” or volunteers to bridge dissemination and follow-up support (39, 40); a UK study showed that recruiting connectors from diverse ethnic and faith communities can bridge the gap between health systems and “hard-to-reach” populations, improving awareness of and trust in EOL services (40).

System integration also means embedding gamified education within wider health policy and professional training. An American Academy of Nursing expert panel called for palliative competence to be incorporated into the core education and practise of all nurses (41); LGBTQ+ competencies should likewise be required within hospice and palliative medicine fellowship training (29). In low-resource settings such as sub-Saharan Africa, integrating palliative training into primary health-care workforce education is seen as cost-effective and sustainable (42, 43), and scalable, standardised games hold real potential here. Yet a “one-size-fits-all” model must be avoided: a South African study found that frontline implementers integrating palliative care into primary care reported low confidence, staff shortages, and limited training, underscoring the need for adaptation to local resources (44).

Three practical questions determine whether such games are sustainable in routine practise: who funds them, how often they are used, and what they cost clinicians in time. Development and culturally specific adaptation are expensive, and this burden cannot fall on individual departments alone. Realistic financing models include public-health and health-system budgets, investment by governments and professional bodies as part of workforce-competence mandates, competitive research and philanthropic grants, and, in particular, open-licenced or shared “core-plus-adaptation” platforms in which many sites reuse a common engine and fund only their local cultural content, spreading cost across a consortium. Dosing matters as well. Rather than a single exposure, evidence on skill retention favours brief, repeated play with periodic refreshers, for example annually, and a facility may choose to link scenario completion or demonstrated competence to onboarding, continuing professional development credit, or local credentialing and privileging. Finally, the clinician time involved must be accounted for explicitly. Protected time within working hours requires backfill so that patients and cases remain covered, whereas reliance on unpaid personal time is inequitable and unsustainable, falling hardest on frontline and lower-paid staff; the time burden is therefore itself an equity consideration.

## 6 Controversies, gaps, and future directions

Two debates recur throughout this literature. The first concerns the tension between gamification and serious subject matter. One camp holds that, since play centres on “fun,” gamifying death, suffering, and grief may seem frivolous or disrespectful to patients and families (4); the other argues that the psychological distance and safe space afforded by play let learners explore emotionally complex scenarios more freely, and that this “safe risk-taking” is precisely what enables deep learning. A systematic review calls for a theory-driven, rigorous design to balance seriousness and engagement (4). The second concerns simulation fidelity. Because branching games necessarily simplify reality, critics doubt they can capture the uncertainty, incomplete information, and emotional intensity of acute EOL care; proponents counter that even simplified scenarios let learners rehearse transferable communication frameworks and decision processes, provided the design captures the clinical “essence” rather than every detail.

Several gaps in the current evidence base stand out. First, high-quality randomised trials of the long-term effects on practise and patient outcomes are lacking; most studies are single-group pre–post or small qualitative designs with low evidence grades (3, 4). Second, culturally adapted games for specific marginalised populations (LGBTQ+, Indigenous peoples, refugees) are markedly underdeveloped; beyond a few efforts for African-American or Latino groups (11, 12), research on broader cultural groups is almost absent. Third, the “active ingredients” of gamified interventions, decision feedback, role-play, narrative immersion, and social interaction, remain poorly understood, though identifying them is vital to optimisation. Fourth, implementation-science research on how to integrate gamified education sustainably across diverse health and education systems is scarce (38).

Several promising directions follow from this analysis. We should encourage interdisciplinary collaboration spanning educational technology, clinical medicine, public health, sociology and anthropology to co-develop, implement and evaluate culturally adapted interventions; adopt mixed methods that combine quantitative (e.g., randomised controlled trials) with qualitative approaches (in-depth interviews, focus groups) to measure not only effect but mechanism and context; advance participatory design and community-based participatory research so that content is co-created with target communities rather than imposed by experts; harness emerging technologies such as AI-driven adaptive learning to tailor difficulty and content dynamically to each learner; and extend the games’ reach from professional to public education, making them a lever for raising societal death literacy and advancing health equity (13, 34, 35).

## 7 Conclusion

**Figure 1:**
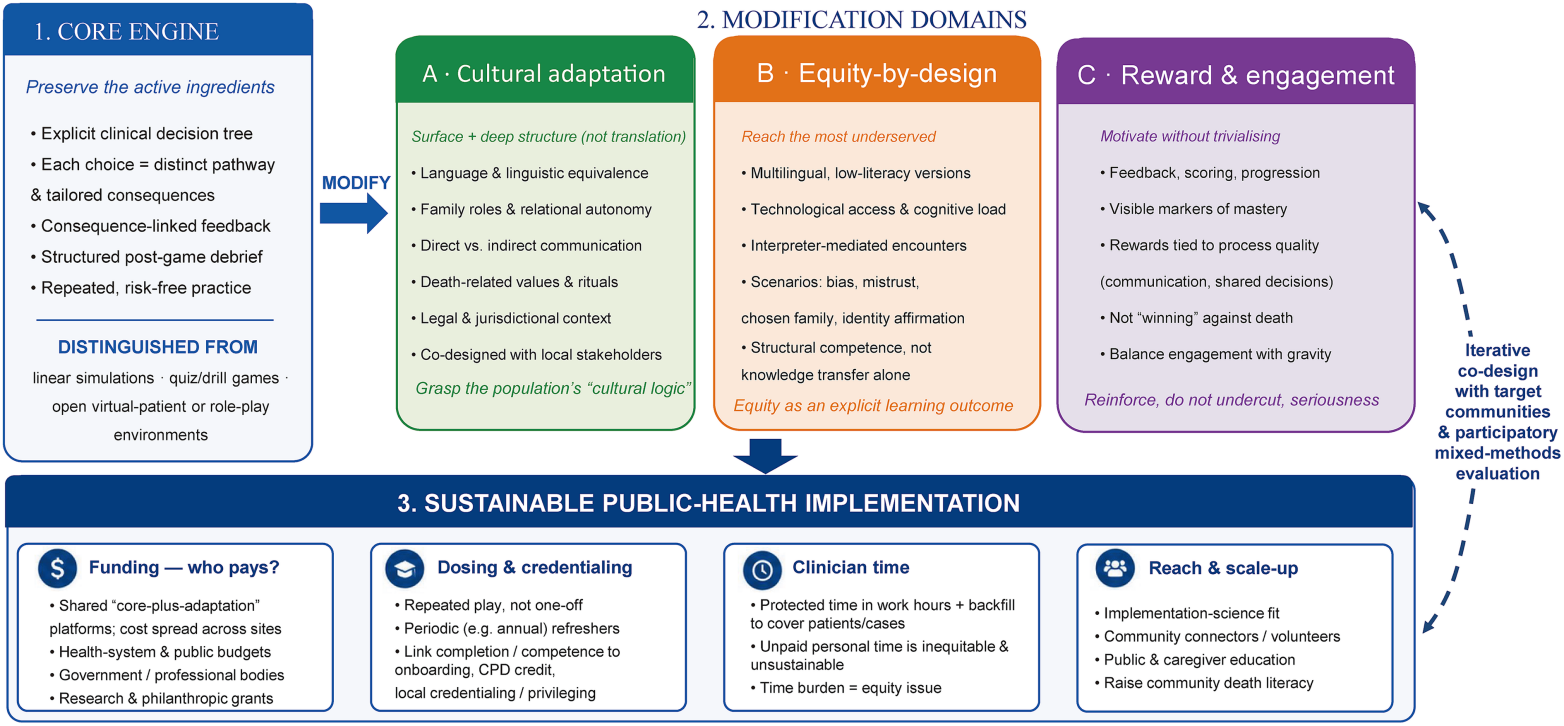
Key elements of branching-scenario game modification for equitable acute end-of-life care education. The core engine of branching-scenario games, including an explicit clinical decision tree, consequence-linked feedback, structured post-game debriefing, and repeated risk-free practise, is preserved while adaptations are introduced across three domains: **(A)** cultural adaptation of surface and deep structure; **(B)** equity-by-design approaches to improve accessibility and relevance for underserved populations; and **(C)** reward and engagement mechanisms that reinforce communication, shared decision making, and process quality without trivialising death. Sustainable public health implementation requires attention to funding, dosing and credentialing, clinician time, and community reach and scale-up. Iterative co-design with target communities and participatory mixed-methods evaluation support the overarching goal of equitable and dignified acute end-of-life care for all. CPD, continuing professional development; EOL, end-of-life.

Branching-scenario games show considerable promise in acute EOL care education, particularly for building communication confidence, rehearsing complex decisions, and creating a safe learning environment. That promise is not realised automatically, however; it depends on careful cultural adaptation and a deep commitment to health equity. Converting these games from “universal” to “culturally specific” tools demands a systematic, community-engaged process of redesign attentive to language, values, family dynamics, and structural barriers (Figure 1). Viewed through a public health implementation lens, their use should extend beyond professional education to community empowerment, serving as a lever for public death literacy and equity. Future work must confront current controversies and gaps through interdisciplinary collaboration, rigorous empirical study, and participatory design, so that branching-scenario games evolve from a novel teaching technology into a powerful engine for equitable EOL care and the public health goal of a dignified death for all.

## References

[1] Peeler A Afolabi OA Sleeman KE El Akoum M Gafer N Hammerich A . Confronting global inequities in palliative care. BMJ glob. Health. (2025) 10:e017624. doi: 10.1136/bmjgh-2024-017624, 40379276 PMC12086877

[2] Stanich J Sunga K Loprinzi-Brauer C Ginsburg A Ingram C Bellolio F . Teaching palliative care to emergency medicine residents using gamified deliberate practice-based simulation: palliative gaming simulation study. JMIR Med Educ. (2023) 9:e43710. doi: 10.2196/43710, 37585258 PMC10468704

[3] Bahar VI Cakmak B Bilgehan T Alwawi A Calik A. Effectiveness of serious game activities in raising palliative care awareness among nursing students. Comput Inform Nurs. (2026) 4:e01416. doi: 10.1097/CIN.0000000000001416 41498142

[4] Peek I Harris D Rawlinson F Gallard S. Gamification, game-based learning and serious games in palliative care education: systematic review. BMJ Support Palliat Care. (2026):spcare-2025-006005. doi: 10.1136/spcare-2025-006005, 42049275

[5] DeBoer RJ Uwamahoro P Ndoli DA Rugengamanzi E Mulindabigwi A Bigirimana JB . Adaptation of a serious illness communication training intervention for the Rwandan context. J Pain Symptom Manag. (2026) 71:243–252.e5. doi: 10.1016/j.jpainsymman.2025.10.010, 41135916 PMC12702458

[6] Zhang L Huang YL Wu XQ Liu CY Zhang XL Yang XY . The impact of virtual clinical simulation on nursing students’ palliative care knowledge, ability, and attitudes: a mixed methods study. Nurse Educ Today. (2024) 132:106037. doi: 10.1016/j.nedt.2023.106037, 37976886

[7] Ma Q Wu Y Fang R. Truth-telling and ethical considerations in terminal care: an eastern perspective. Nurs Ethics. (2025) 32:971–9. doi: 10.1177/09697330241312376, 39786984

[8] Zhou Y Wang A Ellis-Smith C Braybrook D Feng H Harding R. Implementation, processes and outcomes of advance care planning: a culturally and contextually appropriate programme theory developed in Chinese long-term care facilities. Health Expect. (2025) 28:e70291. doi: 10.1111/hex.70291, 40344363 PMC12061843

[9] Dai X Ning X Lin J Jing J Daubman BR Seow H . Cultural adaptation and pilot testing of a basic palliative care curriculum for practicing physicians and nurses in mainland China. J Palliat Med. (2025) 28:1096–101. doi: 10.1089/jpm.2024.0462, 40117121

[10] Dupont C Smets T Monnet F Eneslätt M Tishelman C Van den Block L. The cultural adaptation of the go wish card game for use in Flanders, Belgium: a public health tool to identify and discuss end-of-life preferences. BMC Public Health. (2022) 22:2110. doi: 10.1186/s12889-022-14523-9, 36397020 PMC9672613

[11] Anderson GT. Let’s talk about ACP pilot study: a culturally-responsive approach to advance care planning education in African-American communities. J Soc Work End Life Palliat Care. (2021) 17:267–77. doi: 10.1080/15524256.2021.1976354 34605361

[12] Torres Blasco N Costas Muñiz R Zamore C Porter L Claros M Bernal G . Cultural adaptation of meaning-centered psychotherapy for Latino families: a protocol. BMJ Open. (2022) 12:e045487. doi: 10.1136/bmjopen-2020-045487, 35379609 PMC8981324

[13] Zuo T Yeap SY Lu G. Navigating the delicate balance of autonomy and harmony: a case study on the cultural adaptation of palliative care interventions in China. BMC Palliat Care. (2025) 24:168. doi: 10.1186/s12904-025-01801-7, 40597130 PMC12211151

[14] Mani ZA. Bridging cultural gaps in end-of-life care: the experiences of international charge nurses in Saudi Arabia. BMC Nurs. (2024) 23:865. doi: 10.1186/s12912-024-02514-7 39609764 PMC11606103

[15] Mobarki K Guo P Mobarki MI Efstathiou N. End-of-life care experiences, attitudes and perceptions of intensive care clinicians in middle eastern countries: a systematic integrative review. Nurs Crit Care. (2025) 30:e70201. doi: 10.1111/nicc.70201, 41047720 PMC12498070

[16] Javier NM. Palliative care needs, concerns, and affirmative strategies for the LGBTQ population. Palliat Care Soc Pract. (2021) 15:26323524211039234. doi: 10.1177/26323524211039234, 34527948 PMC8436312

[17] Rosa WE Roberts KE Braybrook D Harding R Godwin K Mahoney C . Palliative and end-of-life care needs, experiences, and preferences of LGBTQ+ individuals with serious illness: a systematic mixed-methods review. Palliat Med. (2023) 37:460–74. doi: 10.1177/02692163221124426, 36475950 PMC10171330

[18] Haviland K Burrows Walters C Newman S. Barriers to palliative care in sexual and gender minority patients with cancer: a scoping review of the literature. Health Soc Care Community. (2021) 29:305–18. doi: 10.1111/hsc.13126, 32767722 PMC7867658

[19] Sítima G Galhardo-Branco C Reis-Pina P. Equity of access to palliative care: a scoping review. Int J Equity Health. (2024) 23:248. doi: 10.1186/s12939-024-02321-1, 39581966 PMC11587758

[20] Estrada LV Agarwal M Stone PW. Racial/ethnic disparities in nursing home end-of-life care: a systematic review. J Am Med Dir Assoc. (2021) 22:279–290.e1. doi: 10.1016/j.jamda.2020.12.005, 33428892 PMC8128037

[21] Pierce DN. Minority populations and the use of palliative care. Crit Care Nurs Clin North Am. (2022) 34:19–29. doi: 10.1016/j.cnc.2021.11.002 35210023

[22] Boucher NA Johnson KS. Cultivating cultural competence: how are hospice staff being educated to engage racially and ethnically diverse patients? Am J Hosp Palliat Care. (2021) 38:169–74. doi: 10.1177/1049909120946729 32734763

[23] Koffman J Shapiro GK Schulz-Quach C. Enhancing equity and diversity in palliative care clinical practice, research and education. BMC Palliat Care. (2023) 22:64. doi: 10.1186/s12904-023-01185-6, 37271813 PMC10239712

[24] Rivera-Burciaga AR Palacios M Kemery SA. Educating for equity in palliative care: implications of the future of nursing 2030 report. J Prof Nurs. (2022) 42:134–9. doi: 10.1016/j.profnurs.2022.06.012, 36150851

[25] Gazaway SB Barnett MD Bowman EH Ejem D Harrell ER Brown CJ . Health professionals palliative care education for older adults: overcoming ageism, racism, and gender bias. Curr Geriatr Rep. (2021) 10:148–56. doi: 10.1007/s13670-021-00365-7, 34745842 PMC8556773

[26] Aaron SP Supiano K DeSimio S. Voices of care: nurses’ perspectives on end-of-life care for black/African American patients. J Hosp Palliat Nurs. (2025) 27:E33–42. doi: 10.1097/NJH.0000000000001081, 39636105 PMC11708984

[27] Almeida-Godinho M Reis-Pina P. Inclusive palliative care for LGBTQIA+ individuals: a socioecological perspective on barriers and enablers. Palliat Support Care. (2025) 23:e183. doi: 10.1017/S1478951525100898, 41078313 PMC13166272

[28] Chidiac C Grayson K Almack K. Development and evaluation of an LGBT+ education programme for palliative care interdisciplinary teams. Palliat Care Soc Pract. (2021) 15:26323524211051388. doi: 10.1177/26323524211051388, 34708209 PMC8543653

[29] Liantonio J Tapper CX Danielewicz M Spina E Javier NM. A call for the creation of LGBTQ+ competencies for hospice and palliative medicine (HPM) fellowship programs. J Pain Symptom Manag. (2023) 65:e381–5. doi: 10.1016/j.jpainsymman.2022.12.009, 36563866

[30] Bigger SE Obregon D Keinath C Doyon K. Language justice as health equity in palliative care: a scoping review. J Pain Symptom Manag. (2025) 69:269–88. doi: 10.1016/j.jpainsymman.2024.11.012, 39643251 PMC11802314

[31] Dookie SP Martin L. The effect of language discordance on the experience of palliative care: a scoping review. PLoS One. (2025) 20:e0321075. doi: 10.1371/journal.pone.0321075, 40173147 PMC11964263

[32] Kanagalingam G Allen J Chin GH Lee HM. Palliative care and chronic liver disease: barriers to care, health disparities and the role of health literacy. Ann Palliat Med. (2025) 14:353–68. doi: 10.21037/apm-25-15 40769731

[33] Bollig G Rosenberg JP. Public health palliative care and public palliative care education. Healthcare (Basel). (2023) 11:745. doi: 10.3390/healthcare11050745, 36900750 PMC10000744

[34] Bollig G Bauer EH. Last aid courses as measure for public palliative care education for adults and children—a narrative review. Ann Palliat Med. (2021) 10:8242–53. doi: 10.21037/apm-21-762 34353105

[35] Macaden L Broadfoot K Carolan C Muirhead K Neylon S Keen J. Last aid training online: participants’ and facilitators’ perceptions from a mixed-methods study in rural Scotland. Healthcare (Basel). (2022) 10:918. doi: 10.3390/healthcare10050918, 35628055 PMC9141240

[36] Bollig G Safi M Schmidt M Ewald H. Is there a need for cultural adaptation of the last aid course?-a mixed-methods study across the Danish-German border. Healthcare (Basel). (2022) 10:658. doi: 10.3390/healthcare10040658, 35455837 PMC9031265

[37] Perin M Tanzi S Botrugno C Craddock C Menkin E Peruselli C . Translation and cultural adaptation of the go wish game: thinking about personal values to promote advance care planning. J Palliat Med. (2022) 25:1540–50. doi: 10.1089/jpm.2022.0083, 35862002

[38] Van Campe F Chambaere K Pivodic L Gilissen J Pesut B Duggleby W . Systematic adaptation of public health palliative care interventions across settings using ADAPT guidance: methodological learnings from the EU NAVIGATE project. Palliat Med. (2025) 39:460–72. doi: 10.1177/02692163251320507, 40105050 PMC12084666

[39] Ontrakrai N Bailey C Valler T Neilson S. Palliative care training programmes for community volunteers working with children and their families: a scoping review. Front Public Health. (2025) 13:1469854. doi: 10.3389/fpubh.2025.1469854, 40453503 PMC12122434

[40] Wainwright JE Cook EJ Ali N Wilkinson E Randhawa G. Bridging inequities in palliative and end-of-life care: the role and experiences of community connectors. Palliat Care Soc Pract. (2025) 19:26323524251369896. doi: 10.1177/26323524251369896, 41883515 PMC13009750

[41] Rosa WE Buck HG Squires AP Kozachik SL Huijer HA Bakitas M . American Academy of Nursing expert panel consensus statement on nursing’s roles in ensuring universal palliative care access. Nurs Outlook. (2021) 69:961–8. doi: 10.1016/j.outlook.2021.06.011, 34711419 PMC8717680

[42] Beecher C Holmes D. Health care worker education for palliative care in Africa: narrative review. Am J Hosp Palliat Care. (2025) 42:121–8. doi: 10.1177/10499091241239645, 38482832

[43] Rosa WE Ahmed E Chaila MJ Chansa A Cordoba MA Dowla R . Can you hear us now? Equity in global advocacy for palliative care. J Pain Symptom Manag. (2022) 64:e217–26. doi: 10.1016/j.jpainsymman.2022.07.004, 35850443 PMC9482940

[44] Arendse JO Zweigenthal V Gwyther L. Operationalising palliative care integration: experiences of implementers in South Africa. Afr J Prim Health Care Fam Med. (2026) 18:e1–e12. doi: 10.4102/phcfm.v18i1.5221, 41925616 PMC13058540

